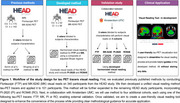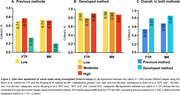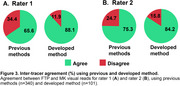# Harmonizing visual readings of tau PET tracers ‐ the HEAD study

**DOI:** 10.1002/alz70862_110845

**Published:** 2025-12-23

**Authors:** Emma Ruppert, Marina Scop Medeiros, Marie R. Vermeiren, Andreia Rocha, Carolina Soares, Pamela C.L. Ferreira, Livia Amaral, Guilherme Povala, Guilherme Bauer‐Negrini, Firoza Z Lussier, Markley Silva Oliveira, Matheus Scarpatto Rodrigues, Rayan Mroué, Bruna Bellaver, Rik Ossenkoppele, Elsmarieke van de Giessen, Joseph C. Masdeu, Dana L Tudorascu, David N. Soleimani‐Meigooni, Juan Fortea, Val J Lowe, Hwamee Oh, Belen Pascual, Brian A. Gordon, Pedro Rosa‐Neto, Suzanne L. Baker, Tharick A Pascoal

**Affiliations:** ^1^ University of Pittsburgh, Pittsburgh, PA USA; ^2^ Alzheimer Center Amsterdam, Amsterdam UMC, Amsterdam Netherlands; ^3^ Houston Methodist Research Institute, Houston, TX USA; ^4^ University of California, San Francisco, San Francisco, CA USA; ^5^ Sant Pau Memory Unit, Hospital de la Santa Creu i Sant Pau, Biomedical Research Institute Sant Pau, Barcelona Spain; ^6^ Mayo Clinic, Rochester, MN USA; ^7^ Brown University, Providence, RI USA; ^8^ Washington University in St. Louis, School of Medicine, St. Louis, MO USA; ^9^ McGill University, Montreal, QC Canada; ^10^ Lawrence Berkeley National Laboratory, Berkeley, CA USA

## Abstract

**Background:**

Tau‐PET provides critical topographic information on tau deposition in the living brain. Integrating research‐based practices into clinical settings through qualitative methods, such as visual reads, is becoming increasingly important. This study aims to compare the performance of existing visual reading methods for [^18^F]Flortaucipir (FTP) and [^18^F]MK‐6240 (MK) and provide preliminary data toward a unified visual reading approach for all tau‐PET tracers using a head‐to‐head dataset.

**Methods:**

The study design and development plans are illustrated in Figure 1. To evaluate previously published visual reading methods, two blinded raters conducted FTP and MK visual reads on 340 participants from the HEAD study. A unified visual reading method was developed and tested on 101 participants, using a composite of adjusted Braak regions to determine 3 stages of severity while harmonizing between tracers. For external validation, the method will be tested on four additional cohorts, each using one of the following tracers: FTP, MK, PI, or RO.

**Results:**

Inter‐rater agreement using previous methods (Figure 2A) showed high Cohen’s kappa values (0.71‐0.77) for the low and high tau burden categories in both FTP and MK visual reads. However, agreement was substantially lower in the non‐AD‐like category, leading to overall agreement rates of 0.55 for FTP and 0.69 for MK. In contrast, the method developed here improved inter‐rater agreement across all categories (0.87‐0.96) and introduced a moderate tau (0.79‐0.83) burden group (Figure 2B), leading to higher overall agreement rates of 0.81 for FTP and 0.87 for MK (Figure 2C). For inter‐tracer agreement (Figure 3), previous methods resulted in discordant visual reads for FTP and MK in 34.4% of cases for rater 1 and 24.7% for rater 2. The developed method reduced this disagreement to 11.9% (rater 1) and 15.8% (rater 2), demonstrating improved consistency across different tracers.

**Conclusion:**

This study demonstrates that previously published visual reading methods produced varying classifications depending on the tracer used. The method proposed here improved inter‐rater and inter‐tracer visual read agreements by developing a unified, tracer‐agnostic approach easy to be used. Importantly, this clinician‐friendly method has the potential for widespread adoption, offering a single harmonized visual reading technique for all tau‐PET tracers.